# Timing of Alcohol and Other Drug Use

**Published:** 2008

**Authors:** Christopher S. Martin

Many Americans who drink alcohol are polydrug users—that is, they also use other psychoactive drugs, such as nicotine, pharmaceuticals, cannabis, and other illicit substances. Polydrug use is a general term that describes a wide variety of substance use behaviors. Different types of polydrug use can be described with regard to the timing of the ingestion of multiple substances. Concurrent polydrug use (CPU) is the use of two or more substances within a given time period, such as a month or a year. Simultaneous polydrug use (SPU) is the use of two or more substances in combination (i.e., at the same time or in temporal proximity) (Grant and Harford 1990*a*). Thus, although all simultaneous polydrug users are, by definition, concurrent users, concurrent users may or may not be simultaneous users. This article describes the functions of SPU for substance users, as well as the measurement, prevalence, patterns, and consequences of SPU.

## Functions of SPU

SPU varies according to the user’s intentions regarding the pharmacological and subjective effects of mixing drugs (Ives and Ghelani 2006; Schensul et al. 2005). Although some SPU may reflect an indiscriminate or haphazard pattern of use, the vast majority of users report a great deal of intentionality regarding their choice of drug combinations (Wibberley and Price 2000). Many experienced substance users demonstrate substantial knowledge of the pharmacology of various drugs and how to combine them to produce certain desired types of intoxication (Merchant and Macdonald 1994; Uys and Niesink 2005).

People often use multiple substances at the same time to produce additive or interactive (i.e., synergistic) subjective drug effects (Wibberley and Price 2000). Perceptions of enhanced or potentiated subjective effects among those who engage in SPU have a pharmacologic basis. Pharmacological evidence exists for the synergistic effects of some drug combinations, such as alcohol and certain classes of sedatives. Another example is the production of the psychoactive compound cocaethylene in those who use alcohol in combination with cocaine (Jatlow et al. 1991). In many cases, more research is needed to determine whether different drug combinations have additive or interactive effects on intoxication and impairment.

Some people also use one drug to reduce or offset some or all of the effects of another substance. In such cases, the user often staggers the timing of ingesting different substances. For example, sedatives may be used to reduce anxiety and allow sleep subsequent to a cocaine binge. A time lag is not always necessary, however, to have one substance counteract or offset the effects of another. For example, some people who are not regular smokers nevertheless smoke while drinking alcohol. The nicotine may help them to maintain a level of alertness, which allows them to better enjoy or prolong an episode of alcohol intoxication (Istvan and Matarazzo 1984). More research is needed regarding intentionality and functionality in various patterns of SPU.

## Measuring SPU

Most substance use measures do not assess combined use and, therefore, can only provide information about CPU and not SPU.^1^ Simultaneous use is more difficult and time consuming to measure than concurrent use (Schensul et al. 2005) and requires the assessment of the temporal proximity of multiple drug use. The specific time frame that ensures combined pharmacologic effects is difficult to determine and depends on factors such as the quantity consumed and the rate of metabolism for each substance. The timeline follow-back technique (TLFB) (Sobell and Sobell 1992) has been adapted to assess the use of alcohol and other drug classes on each of the past 30 days (e.g., Martin et al. 1996*a*), but this provides limited resolution regarding temporal proximity. The SPU Questionnaire (SPUQ) assesses the frequency of use of specific substances within 3 hours of each other (Martin et al. 1992). SPUQ and TLFB measures of the occurrence and frequency of different substance combinations are highly correlated (Martin et al. 1996*a*). Other researchers have developed questionnaires that assess particular alcohol–drug combinations (e.g., Collins et al. 1998; Grant and Harford 1990a,b; McCabe et al. 2006).

## Prevalence of SPU

Alcohol with tobacco undoubtedly is the most common drug combination used in the United States, although their simultaneous use has not been directly assessed in large epidemiological studies. Using data from the National Epidemiologic Survey on Alcohol and Related Conditions (NESARC), Falk and colleagues (2006) found that 21.7 percent of adults (27.5 percent of men and 16.4 percent of women) reported the use of alcohol and nicotine in the past year. Although not measured in the NESARC, it is likely that the large majority of these individuals also used these two drugs simultaneously.

Marked increases in the combined use of alcohol and illicit drugs have occurred over the past 40 years, although little research has focused on the prevalence of specific patterns of SPU. A consistent finding is that SPU is more common in males and among teens and young adults. Marijuana is by far the most common illicit drug used in combination with alcohol in the United States. A national survey conducted in the early 1980s of people ages 12 and older showed that 11 percent of males and 4 percent of females reported combined (i.e., simultaneous) alcohol and marijuana use in the past month. Among 18- to 25year-olds, the percentages were 24 percent for men and 12 percent for women (Norton and Colliver 1988). Using a representative sample of 12th graders studied in the 1990s, Collins and colleagues (1998) reported that 27.6 percent had engaged in combined alcohol and marijuana use in the past year. National survey data collected in 2000 show that 7 percent of those ages 18 and older (8.5 percent of men and 5.5 percent of women) reported past-year simultaneous use of alcohol and marijuana (Midanik et al. 2007).

The prevalence of other alcohol and drug combinations is lower but substantial. Using a representative 1985 adult sample, Grant and Harford (1990*a*, *b*) reported past-year rates of combined use of 1.6 percent for alcohol with sedatives, 3.3 percent for alcohol with tranquilizers, and 4.7 percent for alcohol with cocaine. National survey data from 2000 showed lower rates, with 1.7 percent of adults reporting the combined use of alcohol with an illicit drug other than marijuana in the past year (Midanik et al. 2007). Among 12th-graders in the 1990s, Collins and colleagues (1998) found past-year rates of 2.3 percent for alcohol with sedatives, 6.7 percent for alcohol with stimulants, 5.3 percent for cocaine with other drugs, and 9.9 percent for any substance combination that included an illicit drug other than marijuana. McCabe and colleagues (2006) found that 6.9 percent of a college student sample reported the use of alcohol in combination with nonprescribed pharmaceutical drugs in the past year.

## Patterns of SPU

Those few studies that have assessed the temporal proximity of polydrug use have consistently shown that the majority of concurrent polydrug users also report simultaneous use. This has been found in general population surveys (Grant and Harford 1990; Midanik et al. 2007; Norton and Colliver 1988), among high school and college students (Collins et al. 1998; Martin et al. 1992), among clubgoers (Lankenau and Clatts 2005), and in treatment samples of adults (Martin et al. 1996*a*; Petry 2001) and adolescents (Martin et al. 1996*b*). There are large individual differences, however, in the frequency of simultaneous use. For example, some people report using illicit drugs only rarely while drinking, whereas others use drugs nearly every time they drink (Martin et al., 1996*a*).

The use of three or more substances concurrently and sometimes simultaneously is common among people in addictions treatment. In a treatment sample of adults, Martin and colleagues (1996*a*) found that of those who reported SPU in the past 120 days (61 percent of the sample), about 25 percent consumed alcohol and two illicit drugs in combination, with an average frequency of about once per week, and about 10 percent sometimes drank alcohol in combination with three or more illicit drugs. Similarly, about 17 percent of adolescents in addiction treatment who had an alcohol use disorder reported the use of two or more illicit drugs in combination with alcohol in the past year (Martin et al. 1996*b*).

SPU patterns show age-related differences (e.g., Midanik et al. 2007), regional and ethnic differences (Epstein et al. 1999, 2002), and changing trends over time. Research has highlighted extensive use of alcohol and prescription drugs among college students (McCabe et al. 2006), as well as relatively recent trends of youth and young adults using ecstasy, ketamine, and other “club drugs” in combination with alcohol and other illicit substances (e.g., Lankenau and Clatts 2005; Pedersen and Skrondal 1999). The field needs to continue its surveillance efforts to understand new emerging patterns of SPU, especially among youth.

## Consequences of SPU

SPU can have particularly dangerous consequences because AOD combinations can have additive or interactive effects on acute intoxication and impairment. The majority of deaths attributed to heroin overdose involve significant levels of other drugs such as alcohol or benzodiazepines; opiate levels appear to be similar in both fatal and nonfatal overdoses (Darke and Zador 1996). Similarly, about two-thirds of oxycodone-related deaths were found to involve the use of alcohol and/or other drugs (Cone et al. 2003, 2004). Finally, fatalities and injuries reported to be “alcohol-related” often involve other drug use (Gossop 2001).

Aside from the acute effects associated with intoxication and impairment, little research has examined SPU in relation to health and psychosocial functioning. Although alcohol and tobacco have additive and occasionally interactive effects on health outcomes, such as cardiovascular disease and cancer (Mukamal 2006; Pelucchi et al. 2006), it is unclear whether such effects are related specifically to simultaneous use. Earleywine and Newcomb (1997) found that in a community study of young adults, those who engaged in certain patterns of SPU had more subsequent physical health problems and psychological distress than those who engaged only in concurrent use. Midanik and colleagues (2007) found that compared with concurrent use only, the simultaneous use of alcohol with marijuana, as well as alcohol with other drugs, was associated with increased social problems. Although more research is needed to understand the consequences of SPU, there is little doubt that such behavior represents a major public health problem.

## Footnotes

1 This also is true of the National Epidemiologic Survey on Alcohol and Related Conditions (NESARC).
